# Sensor-driven control strategies for post-stroke shoulder rehabilitation exoskeletons: A systematic review

**DOI:** 10.1016/j.mex.2025.103648

**Published:** 2025-09-28

**Authors:** Madina Karasheva, Anel Saudanbekova, Ardana Utepbergen, Symbat Akkulova, Aibek Niyetkaliyev, Kassymbek Ozhikenov, Assylbek Ozhiken, Chingiz ⁠Alimbayev, Ussen Shylmyrza, Yerzhan Aimukhanbetov

**Affiliations:** a Nazarbayev University, Robotics Engineering Department, Kabanbay Batyr str., 53, 010000 Astana, Kazakhstan; b Satbayev University, Department of Robotics and technical means of automation, Satbaev str., 22, 050013 Almaty, Kazakhstan

**Keywords:** Shoulder exoskeleton, Sensor-driven control, Rehabilitation robotics, Multimodal Sensing

## Abstract

Sensor-driven shoulder exoskeletons are emerging as promising tools for post-stroke rehabilitation, offering scalable, adaptive, and patient-specific motor assistance. This systematic review analyzes 32 studies published between 2015 and April 2025, focusing on the integration of sensor modalities and control strategies in upper-limb exoskeletons targeting the shoulder complex. The review categorizes sensor types, including electromyography (EMG), inertial measurement units (IMUs), force/torque sensors, and kinematic sensors, and evaluates their role in motion tracking, user-intent detection, and feedback regulation. Control strategies are classified into five main groups: force- and admittance-based interaction control, adaptive and assist-as-needed control, human-in-the-loop control, passive support and gravity compensation, and machine-learning-based predictive control. Motor-driven actuation was the most prevalent approach, often paired with advanced control architectures. While multimodal sensor fusion enhances system responsiveness and personalization, most implementations remain in early development or validation stages, with limited clinical deployment. Challenges include sensor drift, bioelectrical signal variability, system complexity, and the need for regulatory approval. The review concludes by highlighting future directions in AI-driven control, wearable sensing, and closed-loop neurorehabilitation. These trends point toward a new generation of intelligent, user-centered exoskeletons capable of delivering high-quality therapy in both clinical and home settings.

## Review Highlights

• The paper systematically examines 32 studies (2015 – April 2025) on sensor-driven shoulder exoskeletons, detailing the use of force/torque, kinematic, EMG, and IMU sensors, as well as multimodal sensor fusion, and classifies control strategies.
• While advanced, multimodal sensing improves personalization and responsiveness, most systems are still in early development or validation phases. Only a small number have reached controlled clinical trials with stroke patients, but results show improved shoulder and elbow mobility, reduced abnormal synergies, and increased functional scores.
• The review highlights critical research gaps in sensor-driven control and outlines future directions for clinical translation of shoulder exoskeletons.

## Graphical abstract

**Figure:**
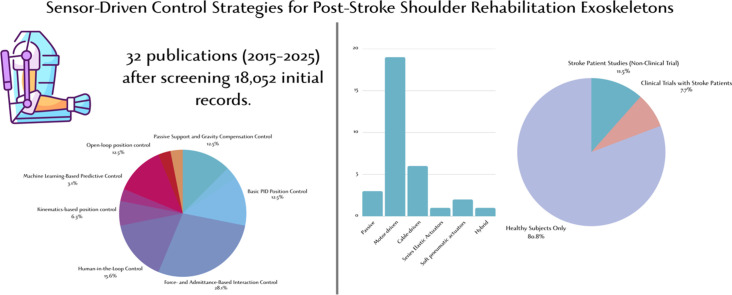

**Specifications table**

**Table:** 

**Subject area**	Engineering
**More specific subject area**	*Rehabilitation robotics*
**Name of the reviewed methodology**	*Shoulder and upper-arm exoskeletons for rehabilitation*
**Keywords**	*Shoulder Exoskeleton; Sensor-Driven Control; Rehabilitation Robotics; Multimodal Sensing.*
**Resource availability**	*The review methods were validated through a comprehensive search of PubMed, Web of Science, Scopus, ScienceDirect, and IEEE Xplore, covering publications from 2015 to April 2025.*
**Review question**	*What sensor modalities and control strategies are employed in post-stroke shoulder rehabilitation exoskeletons? How do sensor combinations influence their clinical application, performance, and future development?"*

## Background

### Introduction

Stroke remains one of the principal sources of long-term disability, and impaired arm-and-hand function is among its most devastating consequences [1]. More than half of all survivors live with some degree of weakness or loss of motor control in the affected limb [2]. Recovery traditionally hinges on labor-intensive, therapist-guided exercises; however, manual therapy is time-consuming, costly, and its quality fluctuates with each clinician’s skill and fatigue [3]. Objective progress tracking is rare, session lengths are short, and the global incidence of stroke is predicted to climb from 3.29 million cases in 2019 to almost 4.90 million by 2030 **-** an increase of roughly 49% [4]. These trends, combined with worldwide shortages of rehabilitation professionals, highlight the need for scalable technological solutions.

Early attempts to meet this need produced hand-coupled end-effector robots that drive the limb through predefined trajectories [[5], [6], [7], [8]]. Although two decades of refinement have made these devices valuable for practicing six-degree-of-freedom (6-DOF) hand tasks relevant to activities of daily living (ADL) [9], they cannot regulate the redundant joints of the arm. Forces applied at the hand alone often provoke abnormal synergies or trunk compensation and can overload proximal joints that lack voluntary control - problems especially detrimental in patients with mild impairments.

Wearable exoskeletons address these limitations by aligning with each anatomical joint and distributing assistance across the limb [10]. Because the upper extremity has about nine functional DOF spanning the shoulder complex, elbow, forearm, and wrist [11], multi-joint exoskeletons can serve both severely and moderately impaired users. Coordinated joint torques suppress compensatory strategies and allow more physiologic ADL practice, making exoskeletons attractive platforms for advanced rehabilitation.

The paper is organized as follows. Section II discusses the biomechanics of shoulder motion relevant to exoskeleton design. Section III outlines the methodology of the systematic review, including the search strategy, screening process, and data extraction framework. Section IV presents an overview of sensor modalities used in shoulder exoskeletons, such as EMG, EEG, force/torque sensors, IMUs, and multimodal sensor fusion. Section V reviews sensor-driven control strategies, including force-based interaction control, adaptive and assist-as-needed schemes, human-in-the-loop approaches, and machine-learning-based control. Section VI summarizes clinical validation studies and outcomes, ranging from healthy-subject trials to early-stage clinical testing. Section VII highlights the key challenges and limitations in the field. Section VIII outlines future directions, including AI integration, wireless sensing, and closed-loop neurorehabilitation. Section IX provides a broader discussion of the findings, and Section X concludes the paper.

### Biomechanics of shoulder motion

**Fig. 1:**
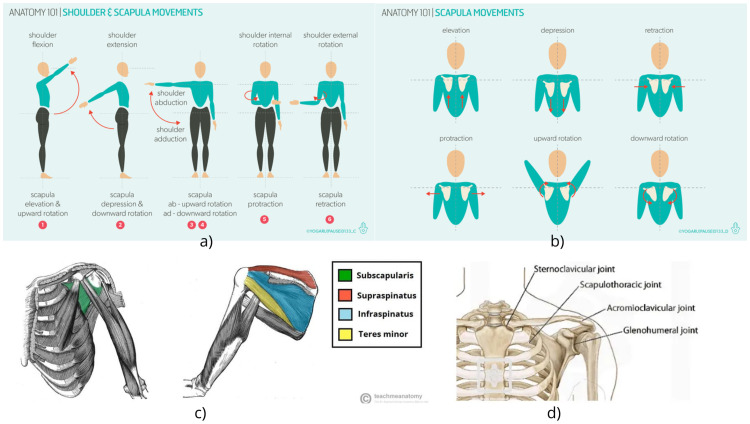
Biomechanical aspects of shoulder movement and muscle anatomy [[15], [16], [17]].

The human shoulder complex is a highly mobile yet vulnerable structure that enables a vast range of motion. It consists of the humerus, scapula, and clavicle and is stabilized by four joints shown in Fig. 1d: the glenohumeral, acromioclavicular, sternoclavicular, and scapulothoracic joints [12,13]. The glenohumeral joint, or ball-and-socket joint, is especially critical due to its broad humeral head and shallow glenoid cavity. This structural feature makes the shoulder inherently unstable and in need of both static and dynamic stabilizers to maintain its position during movements [12,14].

The rotator cuff muscles shown in Fig. 1c, including the supraspinatus, infraspinatus, teres minor, and sub-scapularis, are responsible for the majority of the dynamic stability of the shoulder. These muscles, being close to the center of rotation, ideally contribute to mobility and stability by controlling the rotation of the humeral head and preventing excessive translation of the humeral head within the glenoid cavity [13]. For instance, infraspinatus and teres minor cause external rotation, and subscapularis facilitates internal rotation. The main movement of the deltoid is antagonized by the stabilizing action of these muscles, so it ensures the coordination of movements [13].

Shoulder movements displayed on Fig. 1a and Fig. 1b, such as adduction, abduction, flexion, extension, and rotation, are caused by specific groups of muscles. For example, as indicated in Table 1, abduction is conducted by supraspinatus, middle deltoid, and serratus anterior, while adduction is conducted by teres major, pectoralis major, and latissimus dorsi [12,14]. The biomechanical role of these muscles and their contribution to joint stability need to be understood for injury prevention and designing effective rehabilitation strategies for shoulder pathologies [12,13].

**Table 1:** Shoulder Muscles Involved in Each Movement [16].

Movement	Shoulder Muscles
Abduction	Supraspinatus, Middle Deltoid, Serratus Anterior
Adduction	Teres Major, Pectoralis Major, Latissimus Dorsi
Flexion	Anterior Deltoid, Pectoralis Major, Coracobrachialis
Extension	Posterior Deltoid, Latissimus Dorsi, Teres Major
Internal Rotation	Anterior Deltoid, Teres Major, Subscapularis, Pectoralis Major, Latissimus Dorsi
External Rotation	Posterior Deltoid, Infraspinatus, Teres Minor

Shoulder muscles and joints provide joint stability, range of motion, and safe execution of upper limb movements. Understanding such biomechanical principles is relevant to the clinical assessment of the rehabilitation device design.

## Method details

### Search process description

#### Search strategy

To identify relevant clinical studies on upper limb exoskeletons, a systematic search was conducted across five major academic databases: PubMed, Web of Science, Scopus, ScienceDirect, and IEEE Xplore. The search focused on keywords such as “upper limb”, shoulder, elbow, hand, wrist in combination with exoskeleton, and included filters related to rehabilitation, clinical trials, and clinical studies.

Each database was filtered to include only specific article types such as clinical trials (all phases), observational studies, technical reports, and case reports. Articles were limited to the English language and were published between 2015 and April 2025. Subject areas were restricted to relevant fields, including medicine, engineering, computer science, and neuroscience. Irrelevant articles, such as those focused on lower limbs or industrial robots, were excluded.

Before applying filters, a total of 19,052 articles were retrieved:

• PubMed: 1386 (Keyword: upper limb OR Shoulder OR Elbow OR Hand AND Exoskeleton)
• Web of Science: 4461 (Keyword: ("upper limb" OR shoulder OR hand OR elbow OR wrist) AND (exoskeleton OR orthosis) AND rehabilitation AND (clinical tests OR clinical trials))
• Scopus: 1857 (Keyword:
  (TITLE (upper AND limb AND exoskeleton) OR TITLE (hand AND exoskeleton) OR TITLE (wrist AND exoskeleton) OR TITLE (elbow AND exoskeleton) OR TITLE (shoulder AND exoskeleton)) AND PUBYEAR > 2014 AND PUBYEAR < 2026 AND (LIMIT-TO (SUBJAREA, "ENGI") OR LIMIT-TO (SUBJAREA, "COMP") OR LIMIT-TO (SUBJAREA, "MEDI") OR LIMIT-TO (SUBJAREA, "MATH") OR LIMIT-TO (SUBJAREA, "NEUR") OR LIMIT-TO (SUBJAREA, "MATE") OR LIMIT-TO (SUBJAREA, "HEAL") OR LIMIT-TO (SUBJAREA, "ENER") OR LIMIT-TO (SUBJAREA, "MULT")) AND (LIMIT-TO (DOCTYPE, "ar") OR LIMIT-TO (DOCTYPE, "cp")) AND (LIMIT-TO (LANGUAGE, "English")) AND (EXCLUDE (EXACTKEYWORD, "Robotic Arms") OR EXCLUDE (EXACTKEYWORD, "Artificial Limbs") OR EXCLUDE (EXACTKEYWORD, "Lower Limb") OR EXCLUDE (EXACTKEYWORD, "Agricultural Robots") OR EXCLUDE (EXACTKEYWORD, "Arthroplasty") OR EXCLUDE (EXACTKEYWORD, "Functional Electric Stimulation") OR EXCLUDE (EXACTKEYWORD, "Functional Electrical Stimulation") OR EXCLUDE (EXACTKEYWORD, "Electrotherapeutics")) AND (LIMIT-TO (SRCTYPE, "j") OR LIMIT-TO (SRCTYPE, "p")) AND (LIMIT-TO (PUBSTAGE, "final")) AND (LIMIT-TO (OA, "all")))
• ScienceDirect: 2693 (Keyword: ("upper limb" OR shoulder OR hand OR elbow OR wrist) AND (exoskeleton OR orthosis) AND rehabilitation AND clinical trial)
• IEEE Xplore: 8655 (Keywords: Exoskeleton AND upper-limb OR shoulder OR elbow AND clinical trial AND rehabilitation)

After applying relevant filters, 1268 articles remained. After removing duplicates, the number was reduced to 1178. Articles unrelated to the shoulder region were then excluded, resulting in 248 articles. Finally, after manual screening and excluding studies not relevant to clinical use, a total of 32 articles were selected for final analysis.

#### Article screening process

**Fig. 2:**
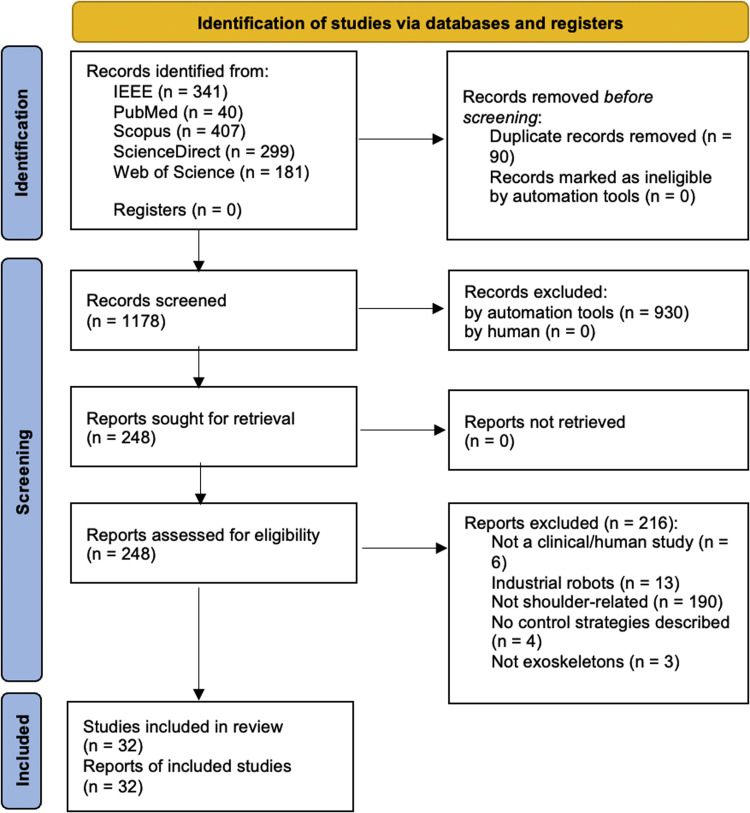
PRISMA Diagram [[18], Fig. 1].

The article screening process followed the PRISMA (Preferred Reporting Items for Systematic Reviews and Meta Analyses) guidelines, and the detailed flow of this process is presented in Fig. 2. After applying database-specific filters and removing duplicates, 1178 articles remained. These articles underwent title and abstract screening, during which non-relevant topics (e.g., lower-limb exoskeletons, prosthetics, industrial applications, or purely mechanical design papers) were excluded. Subsequently, 248 articles that included shoulder or upper-limb (shoulder included) rehabilitation using exoskeletons were identified. The scope was narrowed to only shoulder exoskeletons due to the lack of comprehensive reviews in this specific area; only two relevant articles were identified, published in 2017 and 2023, respectively [19,20]. A manual full-text review was conducted on 248 articles to ensure alignment with the research scope, which focused on the use of sensor-driven control strategies for post-stroke shoulder exoskeletons. During this phase, 216 articles were excluded for the following reasons: they were not clinical or human studies, described industrial robots, were not related to shoulder rehabilitation, did not describe any control strategies, or were not exoskeleton-based systems. As a result, 32 studies met the eligibility criteria and were included in the final systematic review.

#### Data extraction and classification framework

A structured data extraction process was employed to analyze and compare the selected studies. Each article was reviewed and coded using a standardized framework across the following categories:

- Sensor Types: Electromyography (EMG), Inertial Measurement Units (IMU), Force/Torque Sensors, Kinematic Sensors, Multimodal Sensor Fusion
- Control Approaches: Force- and Admittance-Based Interaction Control, Adaptive and Assist-as-Needed (AAN) Control, Human-in-the-Loop Control, Passive Support and Gravity Compensation Control, Machine Learning-Based Predictive Control
- Targeted Limb Region: Shoulder OR Full upper-limb
- Actuation Method: Motor-driven, Cable-driven, Passive (e.g., spring-operated), Soft exosuits
- Clinical Evaluation: Participant group (stroke patients, healthy subjects, etc.), Sample size, Outcome measures (e.g., Fugl-Meyer Assessment, ARAT, ROM, usability feedback), Study setting (laboratory, hospital, home)

#### Risk of bias assessment (ROBIS tool)

We used the ROBIS tool to evaluate the quality of methodology and risk of bias of the reviews included. ROBIS assesses reviews in three stages: (1) eligibility criteria of the study, (2) identification and selection of studies, (3) data collection and study appraisal, and (4) synthesis and findings. Lastly, the instrument gives a general assessment of the risk of bias for each review. All reviews were independently evaluated by two reviewers, and any differences in opinion were discussed.

### Sensor modalities in shoulder exoskeletons

#### Electromyography (EMG)

**Fig. 3:**
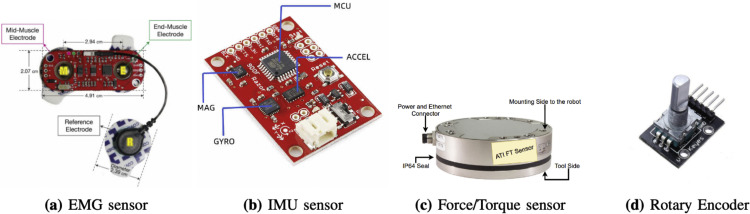
Examples of sensor integration in shoulder rehabilitation exoskeletons: [[22], [23], [24], [25]].

An increasing amount of literature has shown that surface electromyography (EMG) sensors, demonstrated in Fig. 3a, are an essential device for measuring muscle activation of robotic exoskeletons. Objective measurement of neuromuscular involvement plays a key role in post-stroke rehabilitation, where determination of the level of muscular involvement forms the basis of an individualized treatment program and progression. In this regard, the reviewed studies have focused on the muscles of the upper limbs that are most definitive in terms of rehabilitation, that is, the deltoid, biceps brachii, triceps brachii, trapezius, and pectoralis major, using EMG to measure musculoskeletal dynamics. EMG was collected simultaneously with shoulder-elbow kinematics during motor learning tasks involving an exoskeleton that assisted in the CASIA-EXO system. As shown in Table A in supplementary materials, it indicates that joint trajectories and muscle activity are correlated, and therefore the effect of robotic modalities on neuromuscular coordination is clarified [21].

As demonstrated in Table A, the quaternion-based shoulder exoskeleton used EMG sensors to test the hypothesis that optimal trajectories calibrated through kinematic models produced the desired activation patterns in the deltoid and trapezius muscles. This assessment confirmed that system-induced postures have a predictable effect on muscular activity [26]. The self-aligning exoskeleton (NESM-γ) installed electrodes on the anterior deltoid and pectoralis major to characterize user engagement and metrics, and spatial data was measured on electromagnetic field sensors [27]. Simultaneous EMG, joint torque, and orientation measurements provided more information about synchrony between the user and exoskeleton, allowing for a more fine-grained evaluation of rehabilitation performance.

**Fig. 4:**
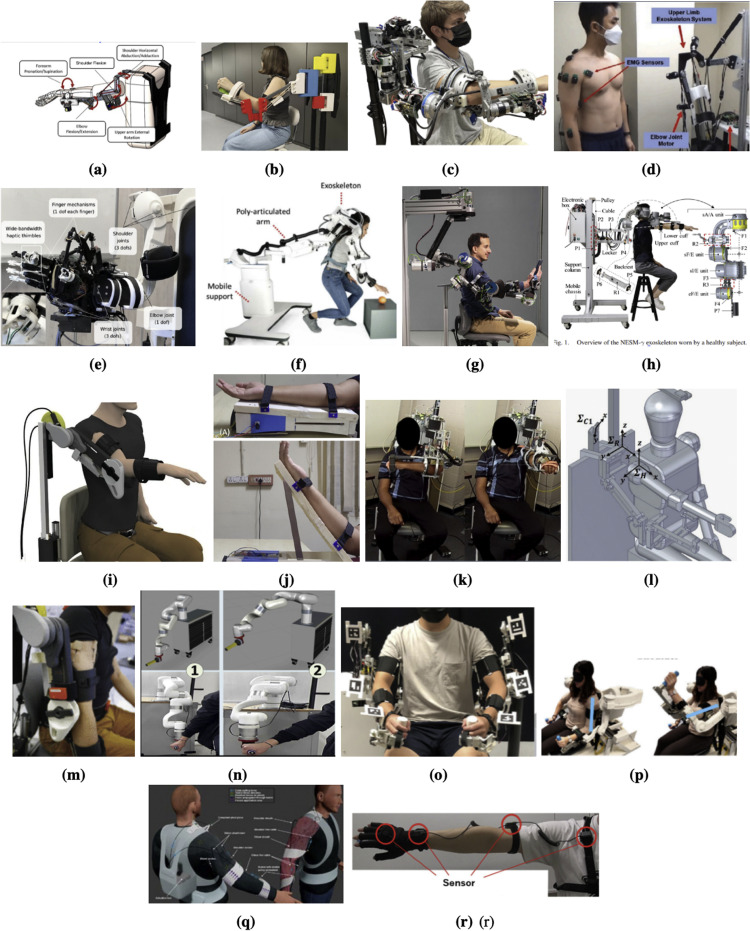
Representatives of Upper Limb (Shoulder) Exoskeletons: [10,26,28,[29], [30], [31], [32], [33], [34], [35], [36], [37], [38], [39], [40], [41], [42], [43]].

Overall, these results demonstrate the utility of surface EMG in the design and testing of upper limb rehabilitation exoskeletons. EMG allowed identification of involuntary muscle movements or co-contraction, providing an objective way of evaluation of motor planning and execution impairment. Another example of the same manner is the study described in [28] demonstrated at Fig. 4i, where EMG sensors were placed on the deltoid and trapezius muscles of stroke subjects. As shown in Table A, these sensors measured upper-arm involvement in repetitive shoulder movements, thus displaying interlimb asymmetries and measuring the effect of gravity compensation in a task-specific training situation.

#### Inertial measurement units (IMUs)

Inertial measurement units (IMU), shown in Fig. 3b, represent a common modality used in upper limb rehabilitation exoskeletons due to their ability to provide constant measurements of segment orientation, angular velocity, and acceleration. An IMU usually combines triaxial accelerometer and gyroscope sensors, and some IMUs also include a magnetometer to provide an estimate of absolute heading. They are particularly well suited to wearable systems and rehabilitation environments where telerehabilitation or home rehabilitation is relevant, since they are relatively compact, have low power needs, and can transmit data wirelessly.

The reviewed exoskeleton systems have used IMUs as independent sources of kinematic information or in sensor fusion systems. The study presented in [44] offers an in-depth report of the accuracy of shoulder-angle estimation with Xsens MTw sensors. The paper shows that the angular errors that are caused by the incorrect orientation of IMU coordinate axes with respect to anatomical planes could generate significant angular differences. A number of calibration methods were explored to mitigate orientation drift and heading misalignment. This demonstrates the importance of exact sensor placement and calibrations in a clinical setting. In this study, the trajectory optimization framework that incorporated quaternions was used to confirm target postures chosen based on their capability to trigger the desired muscle activity patterns. The framework simulated the orientation of the shoulders with quaternion algebra. It showed the viability of the induced poses by comparing the optimized trajectories with the measurements taken based on IMU on test subjects in their reaching movements [26].

The outcomes, therefore, demonstrate the ability of IMUs to be used both as measuring instruments and as a guidance and validation instrument to motion design in exoskeleton kinematic structures. IMUs have been incorporated into the hardware of a number of systems as motion-tracking devices in robots. The IMU signals in the xArm-5 platform, demonstrated at Fig. 4n provided segmental orientation data that complemented the encoder values and a Kinect- based depth camera to increase the motion estimation robustness [39]. IMUs have been used in minimal setups in clinical and pediatric settings where they are used to monitor patients. A single IMU (ENLAZA) mounted on the wrist was deployed in the POWERUP exoskeleton which can be seen at Fig. 4b, a passive and three dimensionally printed pediatric therapy device to monitor hand trajectories during serious games-based training. That configuration allowed therapists to track movement quality and progression over time, even in home-based settings where conventional motion-capture systems are not feasible [30].

#### Force and torque sensors

Force and torque sensors allow quantifying the mechanical interaction between the user and the robot framework. Whereas kinematic sensors monitor the movement and electromyography represents the physiological activation, force and torque sensors help to obtain data regarding the mechanical effort, assistance provided, and the resistance experienced in the course of movement. These sensors play important roles in achieving compliant or impedance control and safety of the user and measuring the physical contribution of both the robot and the human limb. Torque sensors, as demonstrated in Fig. 3c, are commonly installed in joint actuators or placed in series with transmission components to measure the angular moments generated during joint rotation. As an example, the AGREE exoskeleton was designed to measure torque transparently by mounting torsional load cells at the joints. This direct mounting enabled high-fidelity acquisition of joint torque information that enabled real-time impedance control and precise delivery of adaptive assistance depending on patient condition [31]. In a similar way, the BiEXO system employed embedded torque sensors to track assistive torques that the cable-driven actuators of the system provided during shoulder and elbow flexion, to assure proportional and user-responsive support [45].

Abnormal joint behavior was characterized by using torque sensors in systems such as ULIX and CASIA-EXO, and the magnitude of robotic support was measured. The ULIX exoskeleton, demonstrated in Fig. 4k, which included joint torque sensors and electromyography electrodes, allowed the researchers to study neuromechanical coupling in stroke survivors. Particularly, pathological patterns of synergies when shoulder movement caused unintentional elbow flexion were investigated [37]. This joint-level sensing capacity gave a greater understanding of user input, remuneration, and the success of robotic treatment.

A number of systems used force sensors, either at the physical interface between the human and the robot, or in cable-tendon paths. In a cable-driven exoskeleton force sensors were in line with the actuation cables to measure tension during assistance of the limbs. This arrangement allowed precise simulation of the actuation dynamics, especially in cases where compliant elements were used, because they are nonlinearly dependent on tension and length [46]. Force sensors were mounted at the end effector of the xArm-5 robotic platform in order to measure the interaction forces during remote rehabilitation tasks. Such readings allowed two-way control and feedback on safety in physical human-robot interaction, particularly in unsupervised or telerehabilitation settings. The force sensors also proved to be useful in capturing the distributed load in multiple degrees of freedom, and this was equired to adapt robotic support to complex upper-limb trajectories in the system at Fig. 4l [38].

Indirect torque sensing was employed on some systems using series elastic elements or compliant-joint mechanisms. As an example, torque estimation in the system, as demonstrated in Fig. 4p, and in the torque estimation method applied by ANYexo, joint torques were estimated based on deflection measurements over spring-loaded actuators, with encoder readings on the motor and output shafts. It was a simpler method, which did not need costly torque transducers, but did need accurate information on the spring constants and calibration to maintain accuracy under different loading conditions [26].

#### Kinematic sensors

Kinematic sensors form the basis of the design and operation of upper-limb rehabilitation exoskeletons, allowing accurate real-time measurement of joint angles, angular displacements, and trajectories. The workhorse of any motion-based feedback system, these sensors (rotary encoder, optical encoder, magnetic encoder, potentiometers and goniometers) are used to obtain the necessary data to monitor the motion, measure the performance and control the loop.

Rotary encoders, as in Fig. 3d, are usually attached to joint axes or motor shafts in most systems to monitor angular displacement. These sensors are high-resolution, low-latency signals applied in data logging, synchronization, and trajectory control. As an example, the CASIA-EXO exoskeleton was equipped with joint encoders to measure the shoulder and elbow angles during post-stroke motor learning. This enabled the researchers to establish the correlation between the motion of the arm and the pattern of muscular activation and measure the effect of task repetition on the neuromuscular adaptation [21].

Likewise, motor-side and joint-side encoders were fitted in the ULIX and AGREE systems as well. This system allowed estimation of the joint torques through spring deflection and at the same time recorded kinematic data. This dual-encoder architecture in AGREE, which can be seen in Fig. 4c, was critical to the application of high-fidelity impedance control and patient-adaptive smooth assistance [31]. Multiple cable-based mechanisms, such as the Optimized Cable-Driven Exoskeleton, had encoders to track reel movement and pulley rotation, as it is shown in Table A. These were used as indirect proxies of joint angles, which were computed through inverse kinematics of cable routes. Although not as direct as joint-embedded encoders, this solution decreases limb-based sensor weight and allows modular designs of the exosuit [46].

Besides encoders, potentiometers, and goniometers are employed in systems that need economical or wide-angle measurements. The SpringWear exoskeleton at Fig. 4a is a lightweight support frame that was designed to measure compound arm movements by using rotary encoders at the shoulder and elbow coupled with string potentiometers. This combination of setups provided a compromise between the coverage, resolution, and the price [29]. In addition to simple joint tracking, more sophisticated applications of kinematic sensors are in advanced calibration, redundancy, and biomechanical modeling. The commanded and actual positions were compared using high-resolution encoders in the ANYexo platform and the error correction was done in real-time and in high fidelity control. In the same way, during the Self-Aligning Exoskeleton and Multimodal Fusion Study, some encoder information was combined with electromagnetic tracking and electromyography to recreate 3D movement traces and analyze compensatory measures during assisted movement [27,32].

The encoder arrays in [38] and [47] did not only enable single-joint tracking, but also complete-segment re- construction of upper-limb kinematics. The resulting time-series data were analyzed in terms of joint coordination, timing, and smoothness, and have direct applicability to motor recovery assessment. These are measured that led to metrics like range of motion, movement duration, jerk cost, and spatial deviation of task paths [38].

Kinematic sensors are not error-free despite their widespread usage. Disparities may be introduced by backlash, slip, soft-tissue compliance, and structural deflection between measured and actual joint motion. Moreover, encoders do not record the user or robot motion. Consequently, most contemporary systems augment the use of kinematic sensors with electromyography, inertial measurement units, or force sensors as a way of enhancing context and interpretation.

#### Multimodal sensor fusion

With increasing sophistication and clinical ambition of upper-limb rehabilitation exoskeletons to produce a more reliable and richer picture of human-robot interactions multimodal sensing is becoming increasingly dependent. Combining complementary modalities, including electromyography (EMG), inertial measurement units (IMUs), force sensors, torque sensors, and encoders, these exoskeletons eliminate the reliance on any single sensor, thereby increasing the interpretability, adaptability, and personalizability of rehabilitation therapy.

An example is the ULIX exoskeleton, that can be seen at Table A, which uses EMG sensors to capture muscle activity patterns, torque sensors to quantify joint loading, and angular position encoders. The three-pronged sensing strategy allows the system to identify pathological synergies (e.g., inadvertent elbow flexion in shoulder activation) by contrasting muscle activity with mechanical output and joint movement. These understandings help in individualization of assistive measures aimed at suppressing maladaptive patterns and promoting volitional control [32]. The combination of encoders, force sensors and EMG in ANYexo resulted in a high transparency human-robot interface. Each degree of freedom could be controlled with encoder data, and the force sensors measured the forces applied by the user, and the EMG was used to measure voluntary onset of motion. These sensors in combination helped to adjust the level of assistance in real-time, which would be valuable in dynamic rehabilitation exercises, such as reaching or lifting [44].

Strong motion estimation and intention inference is also possible with multi-modal configurations. The Shoulder Exoskeleton, based on Quaternion, incorporated encoder information together with IMUs and EMG to plan posture trajectories that were musculoskeletal feasible and targeted to the desired muscle involvement. Such layered sensing not only permits the tracking of the kinematics of the limbs but also the confirmation that the desired muscle groups were activated as intended [26]. The Self-Aligning Exoskeleton presented a new multi-modal system due to the combination of EMG and electromagnetic tracking, and encoders. In this case, EMG was used to measure user effort, electromagnetic sensors were used to measure scapulohumeral rhythm and compensatory movement, and encoders were used to measure joint trajectories. Such a combination enabled the system to accommodate shoulder movement assistance without compromising the natural joint coupling, which is essential in users who have disturbed motor coordination [27].

Likewise, in the Multi-modal Fusion Study, EMG, force sensors, and kinematic encoders were used in synchronization to record the whole biomechanical and physiological picture. The resulting data allowed not only the delivery of therapy but also the post-hoc analysis of the effort, efficiency, and progression. These systems of data-rich provide clinicians with objective measures of recovery trends and interventional strategies adjustment [32]. Multi-modal sensing is also helpful in telerehabilitation and real-life applications. The xArm-5 system incorporated IMUs, encoders, force sensors, and a Kinect depth camera to assist in motion tracking in remote environments. The redundancy of the sensing types enhanced accuracy when one of the sensors could be failed by occlusion, drift or noise. In [41] platform, that can be seen at Fig. 4p joint encoders and cable tension measurements were fused with IMU during unstructured arm movements to better reconstruct the motion. A number of systems showed that multi-modal sensing does not just apply in robotic control, but also in rehabilitation assessment. The system in [48] integrated EMG, IMUs, and pressure sensors to monitor the interaction between muscular activation, joint orientation, and interaction force during performance of a task. It allowed multidimensional analysis of effort, fatigue, and compensatory movement.

Although the multi-modal systems have advantages, they are associated with greater sensor synchronization requirements, real-time processing needs, and data management. The differences between modalities in timing and inconsistencies in the update rates and interface conflicts should be addressed via well-thought-out communication protocols and software architectures. Moreover, the placement and amount of the sensors can also influence the comfort of users, and they should be carefully incorporated into the ergonomic designs of exoskeletons.

### Control strategies driven by sensors

Effective control strategies are essential for the exoskeletons of upper limb and shoulder rehabilitation to provide efficient, personalized, safe, and effective assistance during therapy. Various approaches have been developed to interpret user intent, modulate robotic assistance, and adapt to the evolving needs of stroke or injury survivors. Below, we outline the major control paradigms commonly applied in upper-limb rehabilitation exoskeletons. Control approaches utilized in different exoskeletons are identified and summarized in Table A. As shown in Table A, most exoskeletons that have reached the stage of healthy-subject testing or clinical trials employ some variation of advanced control strategies. Some systems combine multiple strategies, while others incorporate elements from different approaches that do not fully constitute a hybrid method but are still noteworthy. Moreover, it can be observed that the majority of exoskeletons utilizing basic control types are identified as low-cost systems or in early stages of development. This is expected, as low-cost exoskeletons typically aim to support simpler forms of rehabilitation that can be performed at home. Additionally, there appears to be a clear gap in research on Machine Learning-Based Predictive Control, with only [32] employing this strategy.

#### Force- and admittance-based interaction control

This control strategy relies on measuring interaction forces between the user and the exoskeleton through force or torque sensors. The system interprets these forces to estimate the user’s movement intent. The controller then modulates the exoskeleton’s response by adjusting the admittance, a key parameter that defines how the system responds to an external force [49]. Admittance determines how easily the exoskeleton moves in response to the user’s input forces. A higher admittance means the system moves more freely in response to a given force, while a lower admittance results in stiffer, more resistant behavior [50]. By tuning the admittance parameters, the controller can effectively regulate the compliance of the exoskeleton, ensuring that the assistance provided aligns with the user’s voluntary motor output [51]. This adaptability is particularly advantageous in the early stages of rehabilitation, where patients typically exhibit limited muscle strength and reduced motor control. In such cases, the exoskeleton compensates for the user’s diminished force generation by providing motion assistance in a manner that preserves the user’s sense of agency and promotes engagement in therapy. Furthermore, this strategy enhances safety by minimizing the risk of overexertion or unintended movement, contributing to a more effective and user-centered rehabilitation process.

**Fig. 5:**
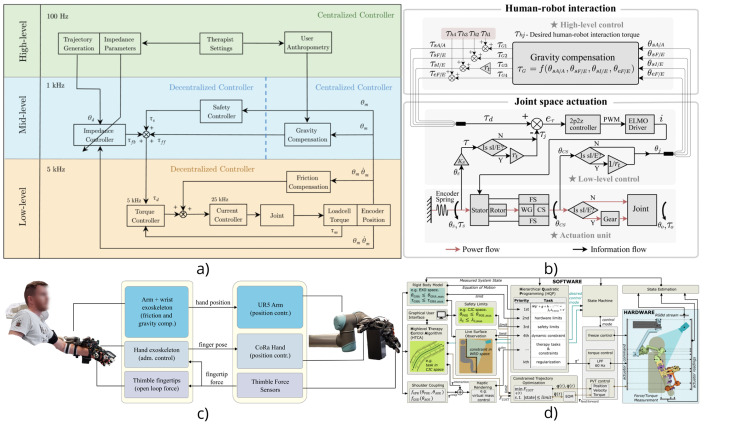
Force- and Admittance-Based Interaction Control in exoskeletons [10,31,33,35].

In [33], the authors implement a Force- and Admittance-Based Interaction Control strategy at the hand exoskeleton level to provide intuitive and responsive teleoperation. Specifically, contact forces measured by sensitive fingertip sensors on the follower robotic hand (CoRa hand), which is presented in Fig. 4e, are transmitted to the leader side and used as reference inputs for an admittance control loop. The control scheme, presented at Fig. 5c, maps the measured forces into velocity commands for the user’s hand exoskeleton, thereby enabling force-to-motion conversion that reflects remote interactions. The admittance controller is realized per finger using strain-gauge sensors mounted at the base of each linear actuator, allowing for individual finger modulation during grasping tasks. To enrich the haptic experience, the system also incorporates a feed-forward tactile feedback pathway, where the same fingertip force signals are high-pass filtered to extract fast dynamic components and drive voice-coil actuators in fingertip-mounted thimbles. This separation ensures that slow, continuous forces are rendered kinesthetically, while rapid transients, such as contact onset, are conveyed as tactile sensations. Together, this dual feedback design supports more natural and effective manipulation by enabling the user to perceive both gross force interactions and fine tactile events, characterizing the system as a clear application of force- and admittance-based interaction control with complementary tactile enhancement.

The AGREE exoskeleton on Fig. 4c implements a multi-layered impedance-based control system, combining low-level torque control with mid-level impedance control and high-level task planning [31]. Each joint features a loadcell-based torque control loop to reject friction and enhance compliance. The mid-level controller uses tun able stiffness and damping gains to shape joint behavior, while a centralized algorithm provides gravity compensation for both the exoskeleton and the user’s arm. The system allows for three training modalities: passive-assisted, active-assisted, and active-resistive. By adjusting the impedance parameters, the controller can smoothly shift from rigid guidance to compliant or resistive behaviors. The admittance-like behavior is replicated at each joint, using feedback torques derived from physical human-robot interaction. Layered control enables transparency during volitional movement and targeted assistance along task trajectories. The control system, which can be seen in Fig. 5a, enables the AGREE exoskeleton to support a wide spectrum of rehabilitation needs. In early-stage rehabilitation, high-stiffness, low-damping control ensures accurate trajectory guidance for severely impaired users. In intermediate stages, reduced stiffness allows more volitional input while still guiding movement. In advanced stages, high damping introduces controlled resistance, challenging the patient to exert more effort, thus promoting neuroplasticity. Moreover, the system’s customizable stiffness-damping space allows for tailored therapy based on a patient’s functional level.

The NESM-γ exoskeleton [35], demonstrated at Fig. 4h, uses a hierarchical control architecture consisting of a High-Level Control Layer (HLCL) and a Low-Level Control Layer (LLCL). The HLCL operates in torque-control mode, where desired interaction torques are specified based on the user’s intention or rehabilitation needs, which is seen in Fig. 5b. These torque commands are interpreted using admittance control principles, where the system dynamically adjusts its mechanical response to external forces exerted by the user. The force-based admittance control allows the robot to remain “transparent” when the user initiates movement, or to assist/resist the motion by modifying joint torques. The LLCL then translates these high-level torque commands into current signals for the actuators, ensuring accurate torque tracking. This interaction model emphasizes patient-in-charge operation, enabling users to drive the exoskeleton actively, while still receiving smooth and safe assistance. By combining series elastic actuators (SEAs) with admittance control, the system enhances compliance and safety, making it particularly suitable for clinical rehabilitation where adaptable and human-centered control is essential.

In the study [10], the authors implement a force- and admittance-based interaction control strategy within a hierarchical control framework to enable safe, compliant, and patient-adaptive rehabilitation. The ANYexo 2.0 exoskeleton can be seen in Fig. 4g. The system architecture is composed of an HLCL and an LLCL. The HLCL operates in torque-control mode, generating desired interaction torques based on user intention and therapeutic goals, while the LLCL translates these torque references into actuator-level motor commands to ensure precise torque tracking. Admittance control is employed to map the interaction forces exerted by the user into corresponding joint motions by modulating the system’s virtual impedance parameters, such as mass, damping, and stiffness. This allows the exoskeleton to exhibit either transparent behavior—where the system passively follows user-initiated movements—or active assistance, in which supportive joint torques are delivered to aid impaired motion. The force-based control scheme thus facilitates a smooth and intuitive interaction between the user and the exoskeleton, adapting to various stages of motor recovery. Overall, this control strategy, displayed at Fig. 5d ensures the system’s suitability for both manipulation tasks and joint-oriented rehabilitation, providing a flexible platform for clinical deployment across different rehabilitation phases

#### Adaptive and assist-as-needed control

The Adaptive and Assist-as-Needed Control framework dynamically adjusts the level of robotic assistance based on the user’s real-time performance [41]. The exoskeleton system implementing this strategy is illustrated in Fig. 4p and comprises actuated joints that support upper-limb movements during rehabilitation tasks. The system monitors user-initiated joint torques and movement accuracy to estimate motor capability and determine the minimum assistance required to complete a task. When the user demonstrates sufficient voluntary effort, the controller reduces robotic support; conversely, assistance is increased when the user is unable to perform the movement adequately. This adaptive modulation is achieved through a performance evaluation index and trajectory tracking error, allowing the system to provide only the necessary support. This not only prevents over-assistance, which may hinder motor learning, but also encourages active engagement, which is essential for effective neurorehabilitation. The control loop continuously updates its internal models to reflect improvements or regressions in user capability, thus personalizing the therapy in real time.

#### Human-in-the-Loop control

**Fig. 6:**
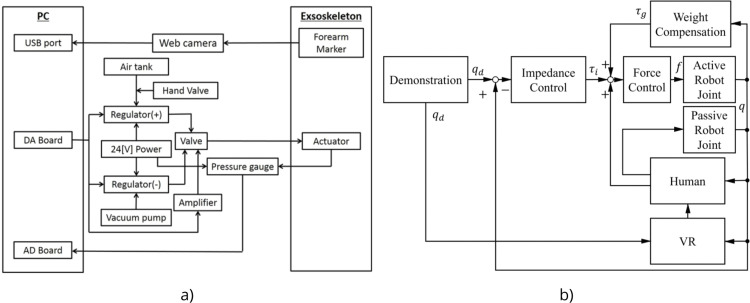
Human-in-the-Loop Control in exoskeletons [38,40].

Human-in-the-Loop control strategy presented in [38] combines vision-based tracking and pneumatic pressure regulation to support adaptive and patient-specific rehabilitation. The upper-limb exoskeleton used in the system, shown in Fig. 4l, features soft actuators that are controlled via a pressure regulation system. A vision-based control law is employed to minimize the positional error between a target and a tracer, enabling real-time trajectory tracking based on the user’s performance. This is achieved through a camera and custom image-processing software, which detects the position of the hand and relays target-reaching accuracy to the control system. The exoskeleton’s soft actuators are driven by a pneumatic system consisting of positive and negative pressure regulators, which are connected to a compressor tank and a vacuum pump, respectively. Each actuator is regulated by a flow valve that switches between positive and negative pressure. Pressure sensors monitor actuator states, and an Analogue-to-Digital converter board transmits these readings to the PC, where control decisions are executed. This multimodal HITL system integrates real-time visual tracking and pressure feedback to dynamically adapt the assistance level during task execution, which can be seen at Fig. 6a. This approach ensures that the robotic system responds to the user’s actual physical interaction and positional accuracy, thereby promoting task-oriented rehabilitation with enhanced user involvement.

The [40] is using HITL control strategy aimed at promoting motor recovery through active user engagement and mirrored motion guidance. The exoskeleton used in this system is shown in Fig. 6b and is designed to support underactuated hand movements for users with impairments on one side. The HITL approach leverages mirrored motion tracking, where a Leap Motion controller captures joint angles of the unimpaired hand and maps them to the impaired hand via the underactuated exoskeleton. This strategy allows the user’s own voluntary movement to directly control the exoskeleton in real time, reinforcing motor intention and encouraging symmetrical motor training. The system, which is seen at Fig. 6b, also integrates Virtual Reality (VR) for task-oriented feedback and uses a 6-axis force/torque sensor at the human-robot interface to monitor interaction forces. By combining real-time user-driven control, visual feedback through VR, and physical compliance via the force sensor, the system maintains the user as an active participant in the loop, ensuring personalized, adaptive, and engaging rehabilitation.

#### Passive support and gravity compensation control

In the [29] control strategy based on Passive Support and Gravity Compensation is presented. The exoskeleton system implementing this approach is shown in Fig. 4a and is designed to support shoulder and elbow movement during rehabilitation exercises. The core idea behind the control strategy is to compensate for the weight of the user’s arm, thereby reducing muscular effort and enabling individuals with severe motor impairments to perform voluntary movements. This is achieved through a counterweight-based mechanical design and a gravity compensation algorithm that provides constant upward torque to balance the arm’s weight. As a result, the exoskeleton minimizes the gravitational load on the user without actively driving movement, allowing the user to initiate and control motion with minimal external interference. This passive assistance is particularly beneficial during early rehabilitation stages or for patients with reduced motor function, as it promotes voluntary motion while preventing fatigue. By reducing the physical burden of arm elevation, gravity compensation control enhances the accessibility and effectiveness of upper-limb therapy in both clinical and home-based settings. Similar gravity-compensation strategies have also been implemented in other exoskeleton systems such as [26,30,34] with the structures of their exoskeletons illustrated in Fig. 4b, Fig. 4f, and Fig. 4m, respectively.

#### Machine learning-based predictive control

**Fig. 7:**
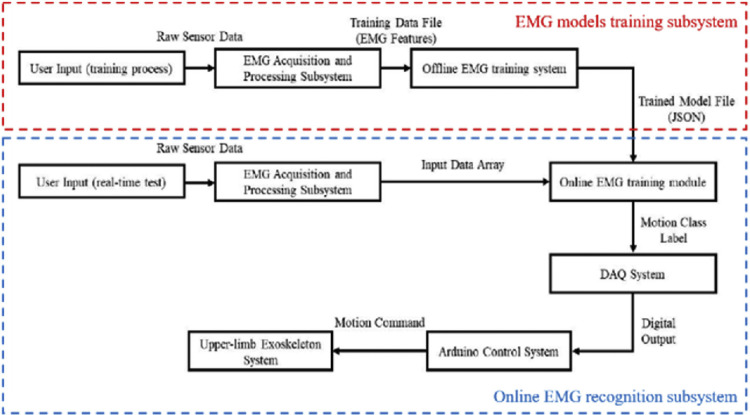
The control framework for Machine Learning-Based Predictive Control [32].

The Machine Learning-Based Predictive Control strategy is implemented in [32] to enhance the adaptability and personalization of rehabilitation therapy. The exoskeleton used in this system is shown in Fig. 4d. The predictive control system relies on multimodal sensor fusion, integrating data from sEMG, EEG, and IMU. A machine learning model is trained to analyze these biosignals and predict the user’s motor intent in real time. Based on the prediction results, the system generates appropriate motion commands for the exoskeleton, thereby assisting the user in executing targeted rehabilitation movements even when voluntary motor output is weak or inconsistent. This system is shown in Fig. 7. By learning from the user’s unique patterns of neural and muscular signals, the model continuously adapts its predictions and improves over time, allowing for highly personalized and intent-driven assistance. This approach supports early-stage rehabilitation, where users may be unable to generate sufficient motion on their own, by proactively interpreting intention and guiding motion accordingly. The integration of machine learning into the control loop makes the system predictive rather than purely reactive, significantly enhancing its responsiveness and therapeutic value. A review of the selected literature reveals that a relatively small proportion of rehabilitation exoskeletons employ EMG-based control strategies. This gap indicates that control paradigms in upper-limb rehabilitation exoskeletons are predominantly grounded in mechanical sensing modalities, such as force, torque, and position measurements, rather than bioelectrical signals derived from the neuromuscular system. Several factors may contribute to this trend. First, EMG signals are inherently noisy and exhibit high inter- and intra-subject variability, particularly in individuals with neuromotor impairments such as stroke, where muscle activation patterns are inconsistent or diminished [32]. Second, accurately interpreting EMG or EEG data to infer motor intent remains a significant technical challenge, especially in patients with reduced voluntary control or atypical muscle synergies. Third, the integration of bioelectrical sensing into medical devices introduces additional regulatory and safety complexities, including concerns related to electrical interference, long-term skin contact, and reliability under variable clinical conditions. As a result, many researchers and developers continue to favor mechanical sensing approaches for their robustness, ease of implementation, and relative simplicity in ensuring safe human-robot interaction. Nonetheless, the underutilization of EMG and EEG control presents a notable gap in the current landscape, underscoring the need for further research into more reliable and clinically viable biosignal-driven control frameworks for assistive robotic technologies.

**Fig. 8:**
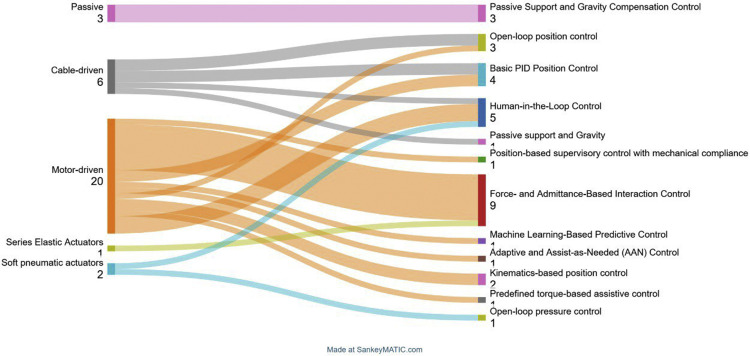
Relationship Between Actuation Mechanisms and Control Approaches in Exoskeletons. Created using SankeyMATIC [52].

Based on the analysis of the table and its visualization in Fig. 8, it is evident that motor-driven systems dominate the field of rehabilitation exoskeletons, offering the most diverse and complex control architectures. Their broad compatibility with both classical and advanced strategies makes them the most versatile actuation platform. The wide range of sensor integrations further enhances their adaptability. In contrast, cable-driven systems tend to employ simpler control schemes, such as open-loop and PID control, which reflect the mechanical limitations and reduced complexity associated with remote actuation. Passive systems rely exclusively on gravity compensation, underscoring their application in low-intervention or assistive-only scenarios. Meanwhile, emerging actuation technologies like Series Elastic Actuators (SEAs) and soft pneumatic actuators are used in a limited number of studies but are typically coupled with intelligent or compliant control strategies, indicating their promise for personalized and adaptive rehabilitation. These trends highlight a shift toward user-centered, sensor-integrated, and adaptive control frameworks, particularly when used alongside compliant actuation.

### Clinical applications and outcomes

The effectiveness of the exoskeleton structure is usually tested on patients or healthy subjects to prove its clinical use. This section groups the clinical trials based on their clinical intensity on three levels. First one is healthy volunteer validation studies, which consist of studies where the participants were non-stroke patients and did not involve a clinical trial. In Table A, this category refers to ”no” and ”no” combination. The next category is stroke patients’ research not involving formal clinical trial design, which is a ”yes” and ”no” combination in the table mentioned above. The third category is clinical trials in survivors of stroke, and it is demonstrated as ”yes” and ”yes” combination in Table 2.

**Table 2:** Key Outcomes from Exoskeleton Studies.

reference	Participants	Stroke Patients	Clinical Trial	Key Outcome
[29]	13	✓	✗	SpringWear significantly improved shoulder flexion, elbow extension, and forearm rotation, with a 19 % increase in reach workspace.
[53]	10 Healthy only	✗	✗	The exoskeleton significantly reduced muscle activity and improved symmetry in bimanual tasks among healthy users.
[33]	7	✗	✗	The system significantly improved task performance, with a higher correct pick-and-place rate and reduced grasping force when haptic feedback was used.
[30]	7 healthy participants	✗	✗	After 8 weekly sessions, participants showed improved hand path efficiency, high task success rates (avg. 88 %), and increased active and passive range of motion in several joints.
[35]	5 healthy subjects	✗	✗	NESM-γ demonstrated precise torque tracking, high transparency, and robust performance across tasks mimicking daily activities.
[34]	none	✗	✗	The Float exoskeleton enabled full shoulder motion in large 3D workspaces, mimicked natural scapulohumeral rhythm, and reduced perceived device weight.
[31]	1 healthy participant	✗	✗	The AGREE exoskeleton demonstrated stable torque control, transparent joint behavior, and a wide range of customizable rehabilitation modalities.
[54]	1 healthy subject	✗	✗	The exoskeleton achieved an 89 % torque reduction at the shoulder and 84 % at the elbow, enabling the use of smaller, cheaper motors.
[45]	1 healthy subject	✗	✗	BiEXO successfully supported upper limb movements with low error (MAE: 5.9 mm) during reaching tasks and showed good control via brain-computer interface.
[32]	28 healthy subjects	✗	✗	The study demonstrated reliable real-time exoskeleton control using EMG signals and machine learning, with SVM achieving the highest online accuracy (90 %).
[47]	11 healthy subjects	✗	✗	Validation showed torque prediction errors under 13 %.
[41]	18 healthy subjects	✗	✗	Using a 6-DoF exoskeleton, the study found that proprioceptive accuracy decreases with multi-joint tasks and is lower at the elbow than the shoulder.
[55]	1 healthy subject	✗	✗	The cable-driven exoskeleton demonstrated safe, flexible, and human-like motion using variable torque mechanisms and EP control.
[36]	20	✓	✓	After 8 weeks, the device group showed greater gains in FMA and AROM scores. TT group: improved from 2.9 ± 0.9 to 10.8 ± 2.1
				RDT group: improved from 2.8 ± 0.8 to 13.6 ± 2.4
				*p* < 0.05 — significant improvement in both groups, with the RDT group showing greater gains
[56]	4 healthy subjects	✗	✗	EIKPE significantly improved joint angle estimation accuracy compared to standard Exoskeleton encoder data, with RMSE and ROM errors reduced by 50–60 %.
[28]	10	✓	✓	Robot-assisted therapy using an optimized assist rate improved shoulder flexion, reduced abnormal elbow flexion, and increased FMA scores in patients with severe upper-extremity paralysis. Fugl–Meyer Assessment (FMA-UE, shoulder/elbow/forearm part)
				Significant improvement:
				Pre: 14.8 ± 5.2
				Post: 15.8 ± 5.4
				*p* = 0.047
				Kinematic outcomes
				Max voluntary shoulder flexion angle:
				Pre: 73.7° ± 34.2
				Post: 84.9° ± 39.0
				*p* = 0.047
				Elbow flexion ratio during shoulder elevation (AUC %):
				Pre: 83.3 ± 6.6
				Post: 88.5 ± 5.0
				*p* = 0.005
[57]	3 post-strokes	✓	✗	The proposed algorithm accurately estimated upper limb joint angles using a single accelerometer, with ∼3.5° RMSE.
[42]	4 healthy subjects	✗	✗	The exosuit reduced muscle activation by up to 26.36 % during elbow flexion and improved trajectory stability, especially in loaded tasks.
[40]	15 healthy subjects	✗	✗	The proposed framework improved motor coordination in an asymmetric bimanual VR task by combining joint-level robot assistance with real-time visual guidance.
[58]	11 healthy participants	✗	✗	The soft wearable robot significantly reduced deltoid muscle activity in healthy participants and improved shoulder movement in SCI individuals.
[37]	16	✓	✗	Gravity support and ULIX therapy modes improved elbow extension, grip release, and shoulder-elbow coordination in stroke patients.
[43]	3 healthy participants	✗	✗	Bilateral control using functional electrical stimulation enables more accurate and faster task performance compared to unilateral control.
[59]	9 healthy subjects	✗	✗	Harmony showed excellent agreement with mocap in elbow and forearm kinematics, and good agreement in shoulder angles, despite systematic offsets.
[46]	5 healthy subjects	✗	✗	The cable-driven exoskeleton demonstrated good trajectory tracking accuracy under PID and impedance control in passive motion tasks.
[26]	1 healthy subject	✗	✗	The quaternion-based system successfully optimized posture trajectories to induce target EMG patterns.
[21]	10 healthy subjects	✗	✗	The CASIA-EXO system enables real-time adaptation of assistance and resistance based on user performance, enhancing movement precision during upper-limb tasks.

#### Healthy subject validation studies

**Fig. 9:**
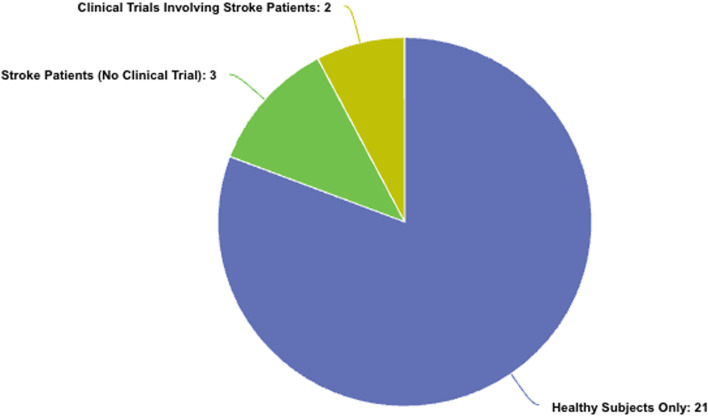
Distribution of Upper Limb Exoskeleton Studies by Participant Type and Clinical Involvement.

As shown in Fig. 9, the majority of clinical validation of the exoskeletons was tested on healthy subjects; specifically, 21 studies were carried out in this format. The results of these studies provide baseline information about device safety, functionality, accuracy of kinematic tracking, patterns of muscle use, and responsiveness of the control under safe experimental conditions.

The NESM-γ, demonstrated at Fig. 4h, is an upper-limb exoskeleton designed for post-stroke motor recovery, using series of elastic actuators (SEAs) for precise torque control [35]. It was tested on 5 healthy subjects to reflect its clinical application. As a result, the exoskeleton demonstrated precise torque tracking, high transparency, and robust performance across tasks that mimic daily activities. The effective transparency was confirmed through parasitic torque remaining low even at higher speeds. The design allows dual-sided use of both arms and aligns with shoulder anatomy, showing strong promise for clinical deployment in stroke rehabilitation.

Parallel to this, an exoskeleton with 5 Degrees of Freedom and performance-adaptive assistance, CASIO-EXO, was used in the experiments on ten healthy subjects, where the system-controlled motor performance adaptively, adjusting the intensity of assistance in real-time [21]. The results showed accurate 3D space trajectory tracking and enhanced movement precision during upper-limb tasks.

The clinical studies on a wearable upper-limb exoskeleton, illustrated in Fig. 4d, with the integration of machine learning (ML) algorithms to process electromyographic (EMG) signals involved 28 healthy subjects [32]. The participants were involved in testing a variety of movements such as drinking, putting down a cup, pushing forward, pulling backward, shoulder abduction, shoulder adduction, and resting. Improved real-time motion pattern recognition accuracy was demonstrated, with the highest real-time accuracy being abduction and resting motions.

In this study, the exoskeleton involves a novel optimization method involving quaternions to generate movement trajectories that respect prescribed muscle activation patterns [26]. Although it was tested on only a single healthy subject, the results of the study showed decreased EMG activation error by 50 % and the ability of optimization based on the posture tailored to elicit specific neuromuscular responses.

Although these are not clinical trials, they are useful proof-of-concept studies. They allow developers to optimize hardware ergonomics, balance control algorithms, and define normative performance measures prior to deployment with clinical populations.

#### Stroke patient studies (Non-Clinical trial)

According to Fig. 9, the number of studies with stroke-patients-only is 3, but it still provides valuable information about the functionality of exoskeletons among the population with motor disability. The pilot trial to assess the efficacy of a 3D-printed rehabilitation device for post-stroke individuals targeted the improvement of shoulder and elbow motions. A total of 20 stroke patients were randomly assigned to two groups: the Rehabilitation Device Training (RDT) group and the Traditional Training (TT) group. Both groups received training for eight weeks, with the RDT group utilizing the 3D-printed device and the TT group receiving traditional therapy. Clinical outcomes like Fugl-Meyer Assessment (FMA) and Active Range of Motion (AROM) were recorded pre- and post-training. The results showed that the RDT group presented significantly greater improvement in shoulder and elbow mobility when compared with the TT group, with significant improvement in FMA and AROM scores (*p* < 0.05). The portable, lightweight, and low-cost device was shown to be an excellent method of improving muscle mobility and strength in stroke patients, providing an effective alternative to conventional rehabilitation techniques.

#### Clinical trials

There are only 2 controlled trials in human beings with exoskeletons included in this paper, as indicated in Table 2 with a combination of stroke patients and clinical trials. The technology remains in the stage of transition from research development to application by real patients, and only a few have progressed to systematic, controlled trials with stroke patients.

The first pilot trial evaluates the efficacy of a 3D-printed rehabilitation device for the rehabilitation of stroke patients on shoulder and elbow movements [36]. The trial consisted of 20 stroke patients who were randomly assigned into two groups: the Rehabilitation Device Training (RDT) group and the Traditional Training (TT) group. Both groups were trained for eight weeks, with the RDT group being treated with the 3D-printed device and the TT group being treated with conventional therapy. Pre- and post-course clinical assessments were conducted with the FuglMeyer Assessment (FMA) and Active Range of Motion (AROM). The results indicated that RDT led to significant improvements in mobility at the elbow and shoulder compared to TT, with a significant increase in the FMA and AROM scores (*p* < 0.05). The machine, being light, portable, and cheap, proved to be a valuable tool in increasing muscle strength and mobility in stroke patients and a possible substitute for traditional rehabilitation methods.

The second study involves a clinical feasibility trial to test a shoulder elevation exoskeleton robot with a novel method of muscle synergy indices [28]. Twelve chronic stroke patients received a 10-day intervention that consisted of 100 robot-assisted shoulder elevation exercises. The results were analyzed by examining the electromyogram (EMG) activity of the anterior deltoid and biceps brachii muscles and calculating the co-contraction index (CCI) for detecting abnormal movement. The outcome measures were evaluated by the Fugl-Meyer Assessment for upper extremity function (FMA-UE), shoulder and elbow kinematic movement measurements, and pain questionnaires. Outcome measures demonstrated better shoulder function, voluntary shoulder flexion, and elbow flexion with shoulder elevation, along with reduced shoulder pain.

In conclusion, clinical trials with exoskeletons in rehabilitation report positive results. The rehabilitation device created by 3D printing significantly improved shoulder and elbow mobility and can serve as a viable and cost-effective substitute for traditional therapy. The shoulder elevation exoskeleton robot optimized with the indices of muscle synergy improved the function of the shoulder and elbow and inhibited abnormal motion and shoulder pain. These studies show the potential of exoskeletons for stroke rehabilitation, and the promising findings of these controlled trials contribute to further enhancements of these technologies. However, it’s worth noting that only a minority of the studies involve stroke patients, because many of the exoskeletons are recent innovations that have not yet reached clinical trials. This review paper focuses on newer developments rather than established devices, which explains the limited number of clinical studies in stroke patients.

#### Challenges and limitations

Despite rapid progress, the clinical translation and widespread deployment of sensor-driven shoulder exoskeletons still face considerable barriers. These limitations span from technical and user-related challenges to regulatory and research gaps.

Sensor modalities such as surface electromyography (sEMG) and electroencephalography (EEG) are central to user-intent detection, yet are highly susceptible to motion artifacts, electrode displacement, sweat, and muscle fatigue [32]. In stroke patients, abnormal coactivation and spasticity further degrade the fidelity of the biosignals [37]. These issues jeopardize the robustness of real-time control, especially during dynamic or prolonged tasks [57].

Post-stroke individuals exhibit a wide range of motor impairments and compensatory movement patterns, requiring individualized tuning of control algorithms [31]. This calibration process, often involving trial-and-error, is time-consuming and may need to be repeated as muscle tone or fatigue levels change throughout rehabilitation [37]. Moreover, systems using machine learning-based volitional control must gather extensive user-specific data to train effective models [10].

Sensor-rich exoskeletons integrating EMG, IMUs, and force sensors tend to be complex, expensive, and difficult to maintain [35]. Many systems rely on bulky structures or wired external computers, reducing their usability in home environments [30]. The need for trained personnel to operate or recalibrate devices further limits their scalability [29,54]. Although lightweight and soft robotic systems are emerging [58], challenges remain in balancing mechanical assistance with compact design [42,55].

For clinical deployment, exoskeletons must comply with regulatory frameworks such as CE and FDA guidelines, which require extensive safety validation [10]. Real-time control based on noisy biosignals raises concerns about unintended motions or injury [43]. Several studies emphasized the need for compliant actuators, passive fail-safe modes, or impedance control strategies to ensure user safety in uncertain conditions [46]. Spastic responses in stroke patients can exacerbate risk during joint-level actuation, as highlighted in [60].

Although many exoskeleton studies demonstrate positive outcomes in lab-based trials with healthy subjects [33], relatively few have conducted large-scale clinical trials on post-stroke populations. For example, the ULIX robot showed potential for flexor synergy therapy [37], but long-term results remain unvalidated in broader groups of patients. A promising pilot study using 3D-printed devices was conducted in [36], but its generalizability remains unproven without larger samples and randomized controlled trials.

### Future directions

#### AI and deep learning integration

The development of control strategies of the upper-limb rehabilitation exoskeletons is increasingly focusing on the use of artificial intelligence (AI) and deep learning. Not too long ago, it was found that deep neural networks can represent nonlinear, high-dimensional relationships between sensor data (namely electromyography (EMG), inertial measurement units (IMU), and force signals) and human intentions, thus enabling predictive and adaptive control architectures. Encouraging current trends are hybrid structures that combine biomechanical models with neural networks, allowing such systems to generalize over a wide variety of users with little calibration [61]. AI approaches have also been proven to be useful in the planning of trajectories as well as in the automated classification of movements [26]. It is important to note that quaternion-based optimization methods of targeted muscle activation have been applied to adapt motion trajectories to users. Moreover, deep learning algorithms provide the potential for transfer learning, where learning models that may be trained on a population can be applied and optimized on another population with minimal retraining. The integration of explainable AI also promotes enhanced clinical trust and interpretability, especially when they are implemented as a decision-support system for therapists [27,61].

#### Wearable and wireless sensor systems

Future work should advance shoulder exoskeletons toward fully wearable, wireless configurations by integrating lightweight, textile-based sensors such as inertial measurement units (IMUs), pressure, and force sensors directly into the garment. This has changed with the current availability of surface EMG and IMU sensors that deliver real-time high-fidelity data at the same time, reducing the discomfort and movement limitation of the patient. Systems based on these sensors have already shown the ability to accurately forecast elbow movement in lightweight, wearable systems, paving the way to unsupervised or at-home rehabilitation sessions [59,61]. They also facilitate continuous monitoring of muscle fatigue, joint kinematics, and interlimb coordination, and feed this information into closed-loop feedback systems. Soft robotic exosuits are growing uses of wearable sensor fusion platforms that integrate or combine force, inertial, and biosignal data [58]. Building on early soft robotic prototypes that demonstrated reduced muscular effort with textile pneumatic actuators [62], the next step is to eliminate reliance on external compressors and tethered electronics, moving toward self-contained systems with onboard power, wireless communication, and embedded sensing. This direction will not only improve usability and comfort but also enable continuous monitoring of movement in home and community environments. Controllers should use robust kinematics-based schemes that tolerate sensor drift, garment slippage, and posture changes, while providing transparent anti-gravity support during holds and responsive assistance during elevation/lowering. Prior results show feasibility of textile inflatable actuators for shoulder abduction and multi-joint assistance [63]. The next steps might be self-calibration routines, standardized home-use protocols, and longer trials quantifying usability, durability, and shoulder-trunk movement quality in real-world rehab settings. Regenerative, fully untethered systems are a harbinger of real-world implementation, such as telerehabilitation systems that incorporate cloud-based data analytics and clinician feedback.

#### Closed-Loop neurorehabilitation

Closed-loop neurorehabilitation systems introduce a paradigm shift whereby, in real-time, the assistance varies depending upon the feedback of the user’s performance. Such systems may be able to use signals of physiological measures of engagement, such as electromyography (EMG), electroencephalography (EEG), to track neural involvement and motor intent, and adjust levels of exoskeleton assistance based on these measurements. Very recently, a report has shown that sEMG-based biomechanical models can effectively predict forearm movement with few parameters, lending themselves to real-time incorporation into feedback-based wearable systems [61]. Moreover, such bilateral control systems as the one that combines functional electrical stimulation (FES) with robotic joints are examples of the potential of bidirectional human-machine interfaces [43]. In the future, designs are projected to integrate artificial intelligence into neurofeedback processes, where systems can discover plateaus in motor recovery and automatically adjust training programs. Feedback modalities also individualize level and rehabilitation objectives, such as haptic feedback, muscle co-contraction ratios, and task completion measures [27].

## Discussion

This systematic review highlights the growing importance of sensor-driven control strategies in upper-limb rehabilitation exoskeletons for post-stroke recovery. By integrating multimodal sensing technologies with adaptive control approaches, these systems address the critical need for personalized and intensive rehabilitation, which is often limited in conventional therapy settings. The findings reveal that while significant progress has been made in developing advanced controllers, there are still notable gaps and challenges that must be addressed to fully realize their clinical potential.

Analysis of actuation types across shoulder exoskeletons from the Fig. 8 reveals a clear dominance of motor driven systems, with 19 implementations identified. This prevalence can be attributed to the maturity, precision, and controllability of electric motors, which allow for complex control strategies such as PID, admittance, and machine learning-based algorithms. These systems are particularly well-suited for applications requiring precise joint trajectory tracking, assist-as-needed functionality, and real-time adaptation to user performance.

Cable-driven exoskeletons represent the second most common category, with six occurrences. Their popularity lies in their inherent compliance, lightweight structure, and ability to remotely locate actuators, thereby reducing on arm inertia. Cable systems are advantageous in scenarios where reduced joint load and increased safety are critical, especially in rehabilitation settings.

Passive exoskeletons, though simpler in design, are used in three cases. These systems rely on gravity compensation and elastic elements to support limb movement without active control. Their benefits include low cost, high safety, and suitability for daily support or initial rehabilitation phases, where active assistance is not yet required.

Soft pneumatic actuators and series elastic actuators (SEAs) are represented in two and one cases, respectively. These actuators provide compliance and safety by design, making them ideal for direct physical human-robot interaction. Soft actuators, in particular, offer enhanced comfort and adaptability to body contours, while SEAs provide accurate force control with shock absorption, useful in delicate rehabilitation tasks. Hybrid systems, with only one ex ample, combine multiple actuation methods (e.g., motor-driven and soft glove actuators) to leverage the advantages of each. These designs are emerging as a promising direction for personalized and adaptable rehabilitation technologies.

Overall, motor-driven exoskeletons remain the most versatile and widely adopted due to their adaptability and control precision. However, alternative actuation types continue to play critical roles in specific use cases where cost, compliance, or anatomical compatibility are prioritized.

To test the methodological quality, we used the ROBIS tool, and the findings were summarized in Table B in supplementary material. The majority of reviews, including those of Author A and Author B, were rated as having low risk of bias in eligibility criteria and study selection, but Author C and Author D demonstrate uncertainty in data collection, appraisal and synthesis and hence overall uncertainty in the risk. These results suggest that although evidence base is generally comprehensive and well-methodological the inconsistency in reporting and transparency can lead to a loss of confidence in some conclusions. This trend is similar to the findings in other related rehabilitation technology reviews, in which methodological rigor has also been found to be inconsistent. One of the strongest points of our review is the methodical synthesis of varied sensor-driven approaches along with a rigorous ROBIS assessment; nonetheless, the use of published sources and omission of grey literature can be viewed as the weakness. All in all, the findings indicate the potential of sensor-based control in post-stroke rehabilitation and the necessity of more transparent, high-quality systematic reviews to reinforce evidence in the future.

## Conclusion

This systematic review highlights the current state and evolving trends in sensor-driven control strategies for shoulder rehabilitation exoskeletons. The findings confirm that sensor integration plays a pivotal role in enabling intelligent and adaptive exoskeleton behavior, with force/torque sensors, IMUs, and EMG being the most frequently used modalities. However, the selection of sensors is often driven by trade-offs between accuracy, robustness, user comfort, and real-time feasibility.

Motor-driven systems emerged as the dominant actuation approach, often paired with force- and admittance-based control or human-in-the-loop strategies. These systems provide rich interaction models but require accurate and responsive sensors, particularly in real-time rehabilitation scenarios. Cable-driven systems and passive exoskeletons, while lighter and less complex, generally support only basic control strategies and are less adaptable to patient-specific dynamics.

Despite the widespread use of EMG and IMUs, their full potential remains underutilized. EMG control, though promising for detecting user intent, faces challenges in post-stroke patients due to signal variability and muscle coactivation. Similarly, IMUs, while useful for motion tracking, suffer from drifting and require frequent calibration or fusion with other sensors for reliable performance. Multimodal fusion strategies, though still emerging, show strong promise in combining the strengths of multiple sensor types to enhance accuracy, reduce noise, and support robust control decisions.

From a clinical perspective, the review finds that most studies remain in the healthy-subject or pre-clinical phase, with few progressing to formal clinical trials. While several studies demonstrate positive outcomes in motor recovery and user engagement, a lack of standardized protocols, small sample sizes, and short intervention durations limit the generalizability of results. Moreover, task-specific validation and longitudinal outcome tracking are rarely addressed.

Looking forward, the field is poised to benefit from the integration of machine learning and AI-driven adaptive control, enabling continuous personalization of therapy. Additionally, wearable and wireless sensors can improve usability and long-term monitoring, while closed-loop neurorehabilitation frameworks may further enhance recovery outcomes by combining intention decoding (e.g., EEG/EMG) with real-time feedback.

In summary, while the use of sensor-driven control in shoulder exoskeletons is growing and shows considerable promise, substantial work is needed to translate technological advancements into clinically validated, patient-friendly rehabilitation solutions.

## Declaration of competing interest

The authors declare that they have no known competing financial interests or personal relationships that could have appeared to influence the work reported in this paper.

## Appendix Supplementary materials

Image, application 1
