# Supplementary material for: Sensor-driven control strategies for post-stroke shoulder rehabilitation exoskeletons: A systematic review
Source: MethodsX. 2025 Sep 28;15:103648. doi: 10.1016/j.mex.2025.103648 (PMC12549714; doi:10.1016/j.mex.2025.103648)
Supplement: Supplementary file 1 [file mmc1.docx]

**Supplementary material *and/or* additional information [OPTIONAL]**

**Table A**

Sensors and their use in Shoulder Exoskeletons

| № | Actuation type | Control type | Sensors used | Purpose of the sensor use |
| --- | --- | --- | --- | --- |
| [29] | Passive | Passive Support and Gravity  Compensation Control | Encoders, string potentiometers, video analysis | Trajectory analysis |
| [53] | Motor-driven | Position-based supervisory control with mechanical compliance | 1. Rotary encoders, Potentiometer    2. Current sensors    3. Force-sensing resistors (FSRs) | 1. Exoskeleton motion control 2. Safety Monitoring 3. post-task performance evaluation |
| [33] | Motor-driven hybrid system: rigid exoskeleton with soft glove | Force- and Admittance-Based Interaction Control, Human-in-the-Loop | 1. Encoders (in exoskeleton), Flex sensors (in soft glove), Force sensors / FSRs (Force-Sensing Resistors) 2. IMUs (Inertial Measurement Units), Pressure sensors (in soft glove) | 1. Exoskeleton motion control 2. post-task performance evaluation |
| [30] | Passive | Passive Support and Gravity  Compensation Control | IMU | Post-task performance evaluation |
| [35] | Motor-driven with compliant actuators | Force- and Admittance-Based Interaction Control | 1. Encoders, torque sensors 2. force sensors | 1. Exoskeleton motion control 2. post-task performance evaluation |
| [34] | Passive | Passive Support and Gravity  Compensation Control | None | - |
| [31] | Series Elastic Actuators (SEAs) | Force- and Admittance-Based Interaction Control | 1. Force sensors, encoders 2. EMG | 1. Exoskeleton motion control 2. evaluation of muscle activity |
| [54] | Motor-driven | Basic PID Position Control | Potentiometers | Exoskeleton motion control |
| [45] | Cable-driven | Open-loop position control | 1. Potentiometers 2. Current sensors | 1. Post-task performance evaluation 2. Safety monitoring |
| [10] | Motor-driven | Force- and Admittance-Based Interaction Control | 1. Joint Encoders, Integrated IMUs (in actuators) 2. 6-DoF Force-Torque Sensors, IMUs in 6-DoF sensors | 1. Exoskeleton motion control 2. Post-task performance evaluation |
| [32] | Motor-driven | Machine Learning-Based Predictive Control | Encoders, EMG, EEG, IMU | Exoskeleton motion control |
| [47] | Motor-driven | Force- and Admittance-Based Interaction Control | Force Sensors, Torque Sensors, Encoders | Exoskeleton motion control |
| [41] | Motor-driven | Adaptive and Assist-as-Needed (AAN) Control, Human-in-the-Loop Control, Passive Support and Gravity Compensation Control | Encoders, Motor Current Sensors | Exoskeleton motion control |
| [64] | Cable-driven | Basic PID Position Control | Potentiometers | Exoskeleton motion control, post-task performance evaluation |
| [55] | Cable-driven | Basic PID Position Control | Potentiometers, encoders | Exoskeleton motion control, post-task performance evaluation |
| [60] | Motor-driven with compliant actuators | Force- and Admittance-Based Interaction Control, Human-in-the-Loop Control | Encoders, Torque Sensors, Force Sensors | Exoskeleton motion control |
| [65] | Motor-driven | Basic PID Position Control | Potentiometers, rotary encoders | Exoskeleton motion control, post-task performance evaluation |
| [38] | Soft pneumatic actuators | Human-in-the-Loop Control | 1. Vision System (camera and software), pressure sensors 2. Encoders | 1. Exoskeleton motion control 2. post-task performance evaluation |
| [36] | Motor-driven | Open-loop position control | IMU | Post-task performance evaluation |
| [56] | Motor-driven | Kinematics-based position control | IMU, Goniometers | Exoskeleton motion control |
| [28] | Motor-driven | Predefined torque-based assistive control | sEMG, Motion Capture System, Force Sensor | Post-task performance evaluation |
| [57] | Motor-driven | Force- and Admittance-Based Interaction Control | 1. 6-axis Force Sensor, Joint Encoders 2. RGB Camera, EMG Sensors | 1. Exoskeleton motion control 2. Post-task performance evaluation |
| [42] | Cable-driven | Open-loop position control | Motor encoders | Exoskeleton motion control |
| [40] | Motor-driven | Human-in-the-Loop Control, Force- and Admittance-Based Interaction Control | 1. Stereoscopic camera, rotary encoders, 6-axis Force Sensor 2. IMU, EMG sensors | 1. Exoskeleton motion control 2. post-task performance evaluation |
| [58] | Soft pneumatic actuators | Open-loop pressure control | Load cell, IMUs, Goniometer | Post-task performance evaluation |
| [39] | Motor-driven | Human-in-the-Loop Control | 1. Potentiometers 2. Force sensor, MPU6050 (Accelerometer and Gyro) | 1. Exoskeleton motion control 2. Post-task performance evaluation |
| [37] | Motor-driven | Force- and Admittance-Based Interaction Control | 6-axis Force sensor, Position encoders | Exoskeleton motion control, post-task performance evaluation |
| [43] | Motor-driven (robot side), Functional Electrical Stimulation (FES) on human subject | Human-in-the-Loop Control | 1. Encoders, EMG 2. EMG | 1. Exoskeleton motion control 2. Post-task performance evaluation |
| [59] | Motor-driven | Kinematics-based position control | Perception Neuron (IMU-based motion capture system), Encoders | Exoskeleton motion control, post-task performance evaluation |
| [46] | Cable-driven | Human-in-the-Loop Control | 1. Rotary encoders, Load cells 2. IMUs | 1. Exoskeleton motion control 2. Post-task performance evaluation |
| [26] | Cable-driven | Passive Support and Gravity  Compensation Control | 1. Joint encoder, IMU sensors 2. EMG sensors | 1. Exoskeleton motion control 2. Post-task performance evaluation |
| [25] | Motor-driven | Force- and Admittance-Based Interaction Control, Adaptive and Assist-as-Needed (AAN) Control | 6-DOF Force Sensors, Encoders, sEMG | Exoskeleton motion control |

Note: The numbered items in the fourth column (“Purpose of the Sensor Use”) correspond directly to the sensors listed in the same order in the third column (“Sensors Used”).

**Table B. Tabular Presentation for ROBIS Results**

| **Review** | **Phase 2** | | | | **Phase 3** |
| --- | --- | --- | --- | --- | --- |
|  | **1. STUDY ELIGIBILITY CRITERIA** | **2. IDENTIFICATION AND SELECTION OF STUDIES** | **3. DATA COLLECTION AND STUDY APPRAISAL** | **4. SYNTHESIS AND FINDINGS** | **RISK OF BIAS IN THE REVIEW** |
| Author A | ☺ | ☺ | ? | ☺ | ☺ |
| Author B | ☺ | ☺ | ? | ? | ☺ |
| Author C | ☺ | ☺ | ☺ | ? | ☺ |
| Author D | ☺ | ☺ | ☺ | ☺ | ? |

☺ = low risk; ☹ = high risk;? = unclear risk

**References**

[64] K. Shi, A. Song, and H. Li, “Optimized Design for Cable-Driven Shoulder-Elbow Exoskeleton Robot,” *IEEE Access*, vol. 9, pp. 68197–68207, 2021, doi: 10.1109/ACCESS.2021.3077365.

[65] A. Abane *et al.*, “Mechatronics design, modeling and preliminary control of a 5 DOF upper limb active exoskeleton,” in *ICINCO 2016 - Proceedings of the 13th International Conference on Informatics in Control, Automation and Robotics*, SciTePress, 2016, pp. 398–405. doi: 10.5220/0005984203980405.
